# Supplementary material for: Nanomolar inhibition of SARS-CoV-2 infection by an unmodified peptide targeting the prehairpin intermediate of the spike protein
Source: Proc Natl Acad Sci U S A. 2022 Sep 19;119(40):e2210990119. doi: 10.1073/pnas.2210990119 (PMC9546559; doi:10.1073/pnas.2210990119)
Supplement: Supplementary File [file pnas.2210990119.sapp.pdf]

PNAS
www.pnas.org

Supplementary Information for

Nanomolar inhibition of SARS-CoV-2 infection by an unmodified peptide targeting the prehairpin intermediate of the spike protein

Kailu Yang, Chuchu Wang, Alex J. B. Kreutzberger, Ravi Ojha, Suvi Kuivanen, Sergio Couoh-Cardel, Serena Muratcioglu, Timothy J. Eisen, K. Ian White, Richard G. Held, Subu Subramanian, Kendra Marcus, Richard A. Pfuetzner, Luis Esquivies, Catherine A. Doyle, John Kuriyan, Olli Vapalahti, Giuseppe Balistreri, Tom Kirchhausen, Axel T. Brunger

*Corresponding author: Axel T. Brunger; Tom Kirchhausen
Email: brunger@stanford.edu; kirchhausen@crystal.harvard.edu

This PDF file includes:

Figures S1 to S12
Tables S1 to S2

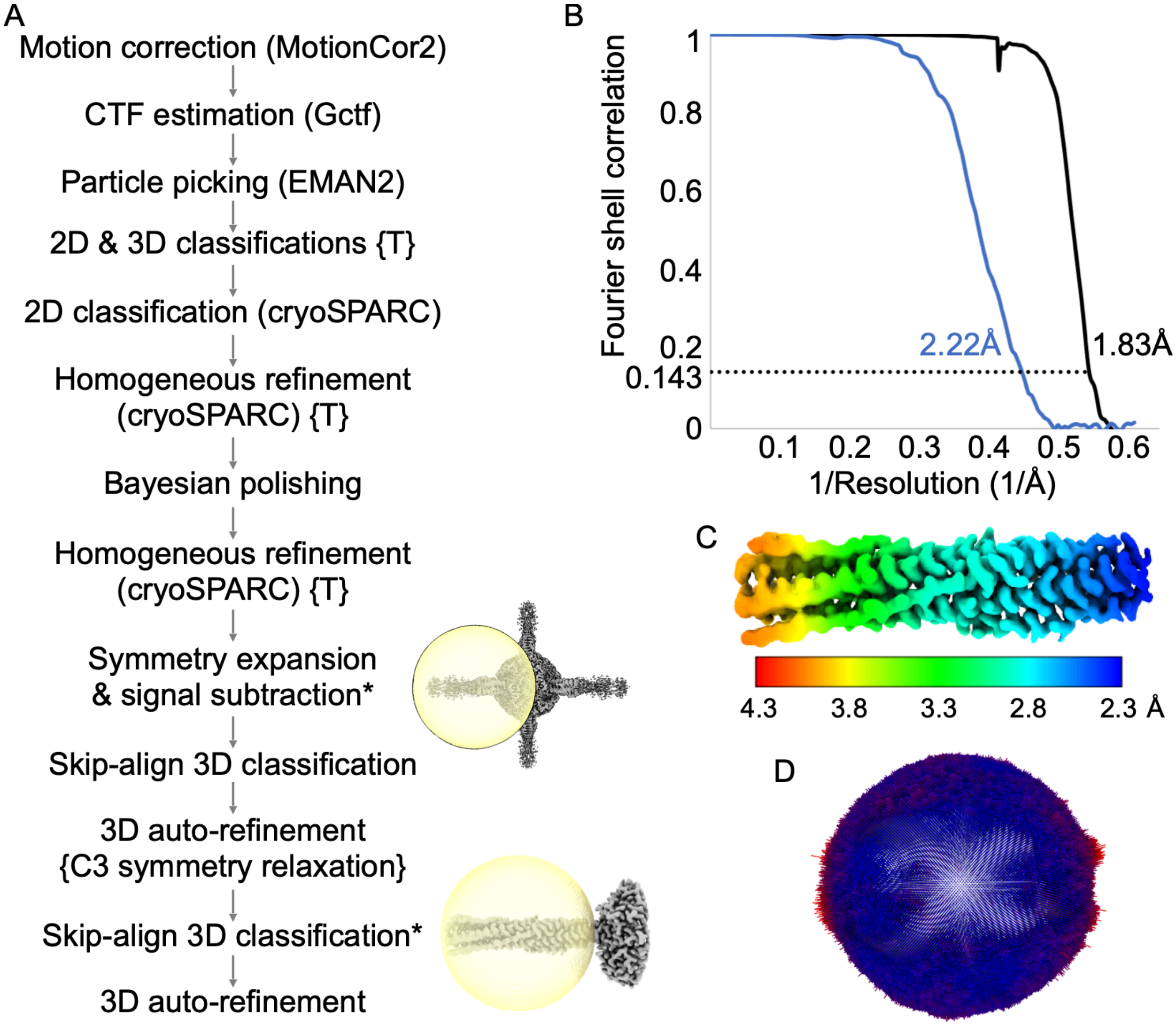

Fig. S1. Cryo-EM structure determination. (*A*) Workflow of cryo-EM data processing. RELION was used for each step unless another program is indicated in parenthesis. Symmetry was imposed or relaxed as indicated in curly brackets. The steps that used a manually generated mask (rather than the default spherical mask in RELION or the default dynamic masks in cryoSPARC) are indicated with a star sign and an image showing the manually generated mask (yellow) and an average map (gray). (*B*) Fourier shell correlations (FSC) of the final refinement step with cryoSPARC (second “homogeneous refinement” using cryoSPARC, black curve) and the final local refinement with RELION (last 3D auto-refinement, blue curve. Note that these FSC calculations were performed using the default spherical mask that covers both the HR1HR2 bundle and part of the scaffold. (*C*) The final reconstruction of the HR1HR2 bundle colored by local resolutions. (*D*) Distribution of the particles' orientations in the final reconstruction, depicted in the same orientation as the map in **c**. The length of each bar is proportional to the number of particles oriented in that direction. The bars are also colored based on the length, with red meaning more particles and blue meaning less particles.

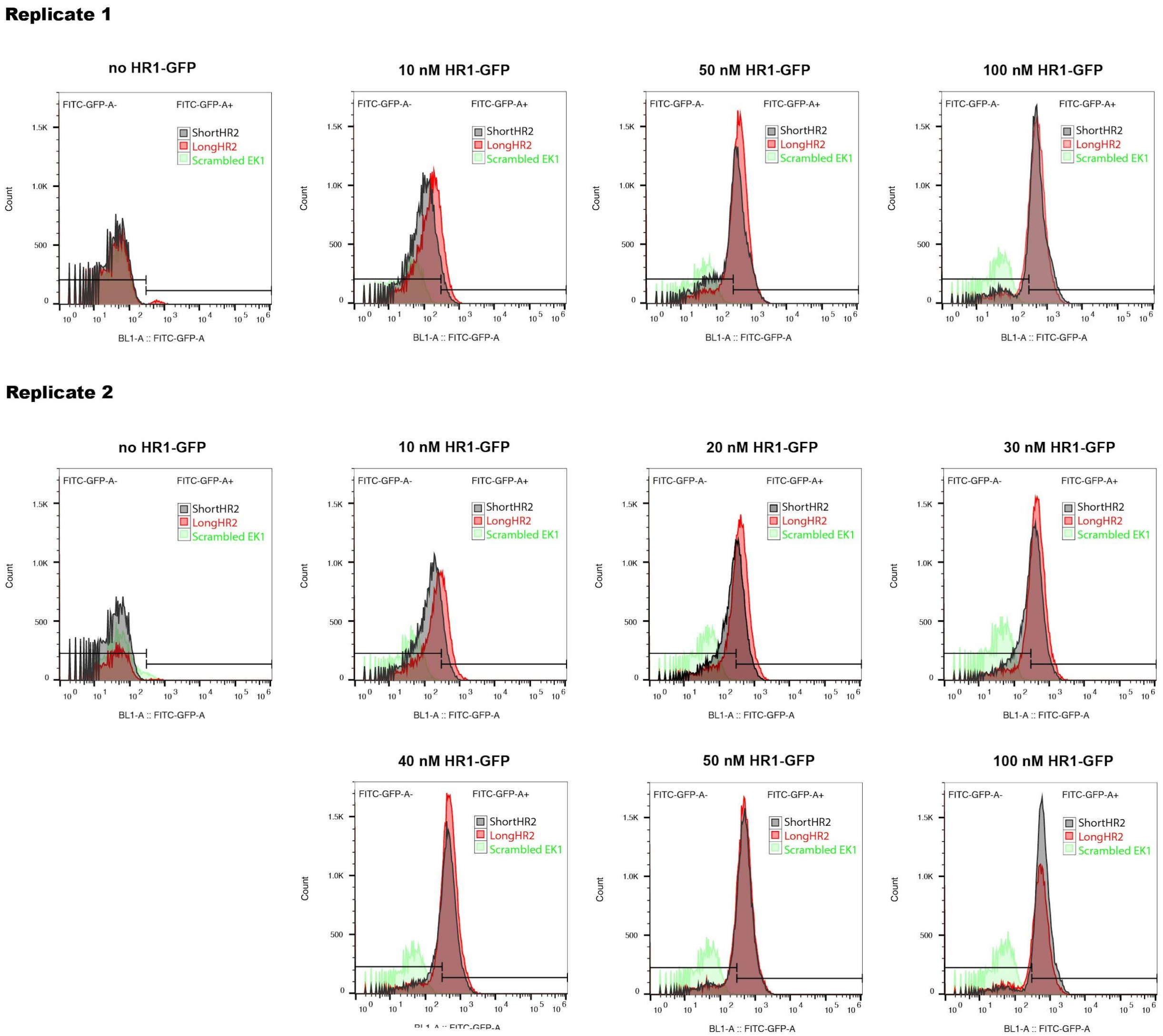

Fig. S2. Flow cytometry histograms of HR1-GFP binding by surface-displayed longHR2_42, shortHR2, and the scrambled EK1 control. Cells displaying either peptide were incubated with different concentrations of HR1-GFP protein and the GFP signal was quantified to compare the binding levels. Regions corresponding to cells were identified using Forward (FSC) and Side-Scatters (SSC), and gates were set to include only these events in the experiments with labeled samples. Gates for the binding analysis were defined using unlabeled cells (no HR1-GFP) and the scrambled EK1 control.

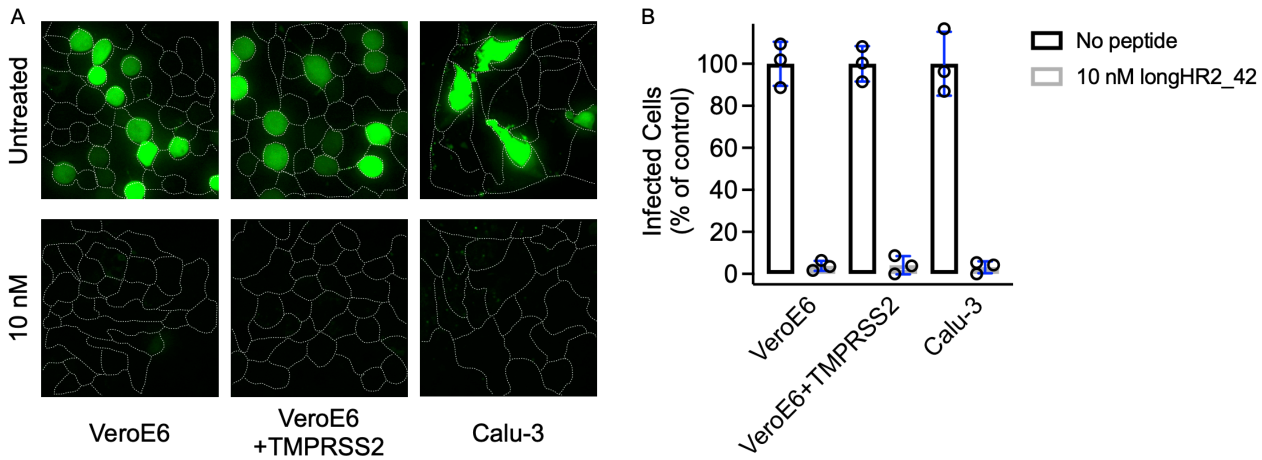

Fig. S3. The strong inhibitory effect of longHR2_42 on VSV-SARS-CoV-2 is independent of cell types with differential expression of host cell proteases (*A* and *B*). Infection (MOI of 0.5) was determined in the absence or presence of 10 nM longHR2 in VeroE6 (cathepsin only), VeroE6+TMPRSS2 (cathepsin and TMPRSS2), and the human lung cell line Calu-3 (TMPRSS2 only). (*A*) Example images are maximum intensity projections of 20 μm with 1 μm z-plane spacing taken on a spinning disc-confocal with eGFP expression allowing for infection to be determined. Cell outlines were obtained from a WGA-Alexa647 membrane stain.

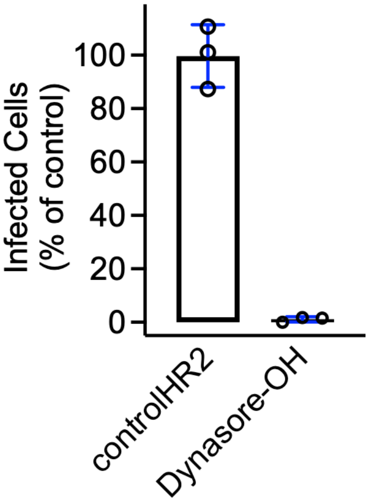

Fig. S4. In the HR2 washout experiment, incubation of cells with controlHR2 had no effect on infection, while keeping dynasore-OH constant throughout the experiment completely inhibited infection.

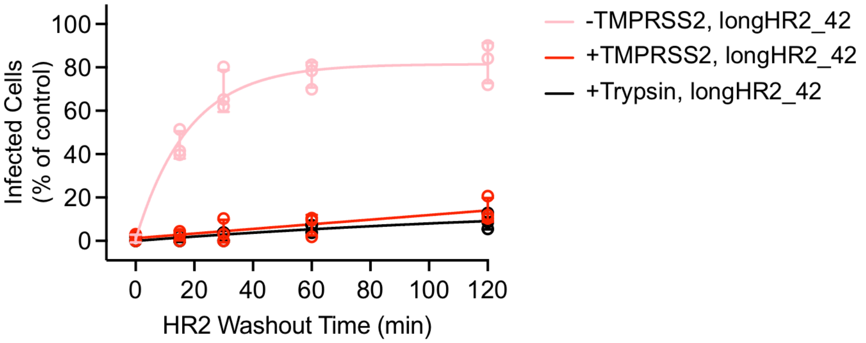

Fig. S5. Trypsin treatment in TMPRSS2-negative VeroE6 cells shows similar inhibition as that of using TMPRSS2-expressing VeroE6 cells in the HR2 washout experiment. The VSV-SARS-CoV-2-Wuh chimera was treated with 1 μM trypsin for 30 min at 37°C followed by 10 μM Aprotinin, a trypsin inhibitor, before adding to the cells. Otherwise, the trypsin experiment was performed with the same condition as that of Fig. 3*E*.

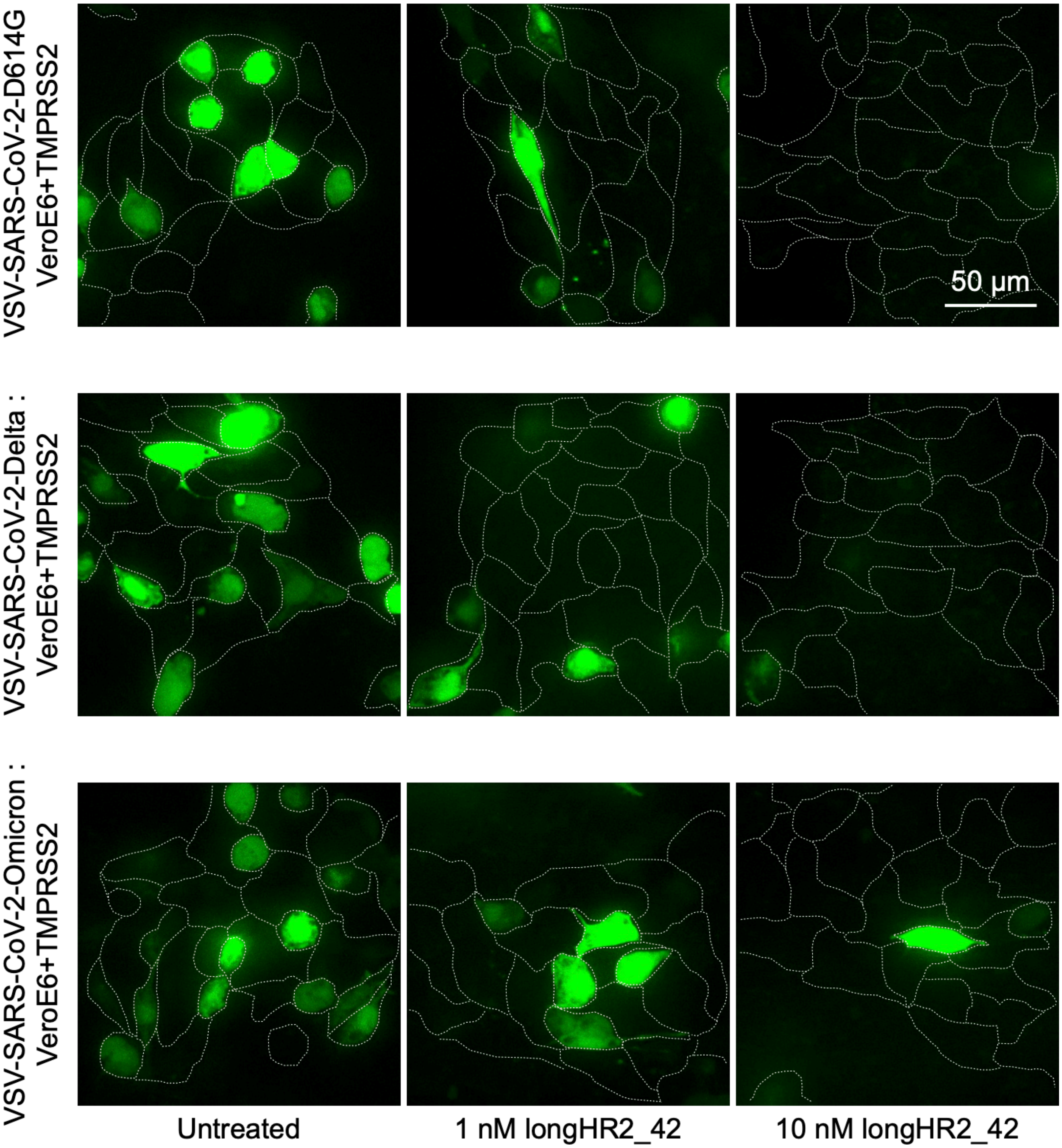

Fig. S6. Example images of the inhibition of VSV-SARS-CoV-2-D614G, -Delta, and -Omicron infection by longHR2_42. Example images are maximum intensity projections of 20 μm with 1 μm z-plane spacing taken on a spinning disc-confocal with eGFP expression allowing for infection to be determined. Cell outlines were obtained from a WGA-Alexa647 membrane stain.

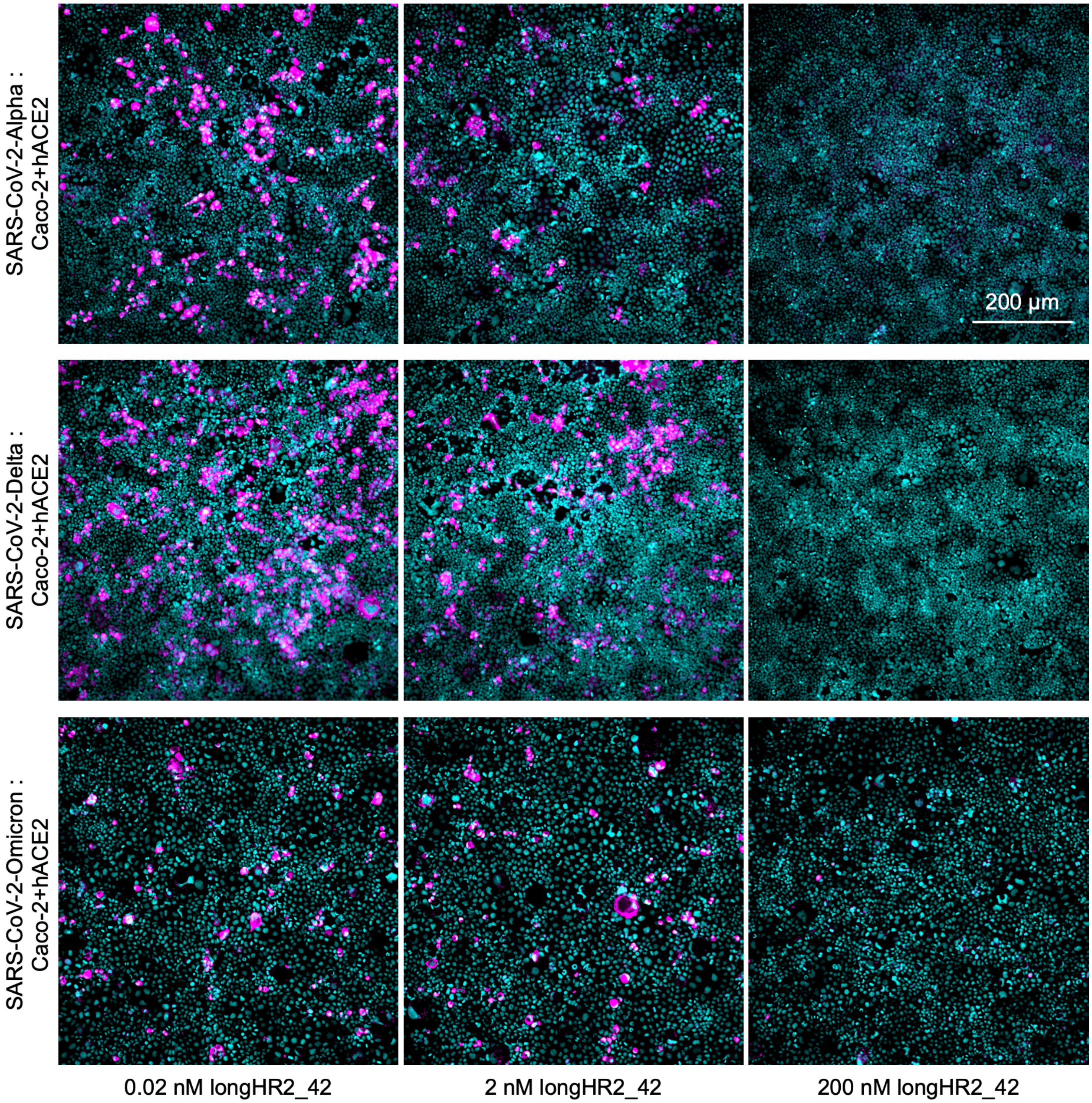

Fig. S7. Example images of the inhibition of SARS-CoV-2-Alpha, -Delta, and -Omicron infection by longHR2_42. The infected cells are detected by an antibody specific for the viral N protein (magenta) and nuclei by Hoechst DNA dye (cyan).

A

HR1

Wuhan	917YENQ KLIANQFNSA IGKIQDSLSS TASALGKLQD VVNQNAQALN TLVKQLSSNF GAISSVLNDI LSRLDKVE988
Alpha	917YENQ KLIANQFNSA IGKIQDSLSS TASALGKLQD VVNQNAQALN TLVKQLSSNF GAISSVLNDI L**A**RLDKVE988
Beta	917YENQ KLIANQFNSA IGKIQDSLSS TASALGKLQD VVNQNAQALN TLVKQLSSNF GAISSVLNDI LSRLDKVE988
Gamma	917YENQ KLIANQFNSA IGKIQDSLSS TASALGKLQD VVNQNAQALN TLVKQLSSNF GAISSVLNDI LSRLDKVE988
Delta	917YENQ KLIANQFNSA IGKIQDSLSS TASALGKLQ**N** VVNQNAQALN TLVKQLSSNF GAISSVLNDI LSRLDKVE988
Omicron	917YENQ KLIANQFNSA IGKIQDSLSS TASALGKLQD VVN**H**NAQALN TLVKQLSS**K**F GAISSVLNDI **F**SRLDKVE988

HR2

Wuhan	1157KNHT SPDVDLGDIS GINASVVNIQ KEIDRLNEVA KNLNESLIDL Q1201
Alpha	1157KNHT SPDVDLGDIS GINASVVNIQ KEIDRLNEVA KNLNESLIDL Q1201
Beta	1157KNHT SPDVDLGDIS GINASVVNIQ KEIDRLNEVA KNLNESLIDL Q1201
Gamma	1157KNHT SPDVDLGDIS GINAS**F**VNIQ KEIDRLNEVA KNLNESLIDL Q1201
Delta	1157KNHT SPDVDLGDIS GINASVVNIQ KEIDRLNEVA KNLNESLIDL Q1201
Omicron	1157KNHT SPDVDLGDIS GINASVVNIQ KEIDRLNEVA KNLNESLIDL Q1201

B

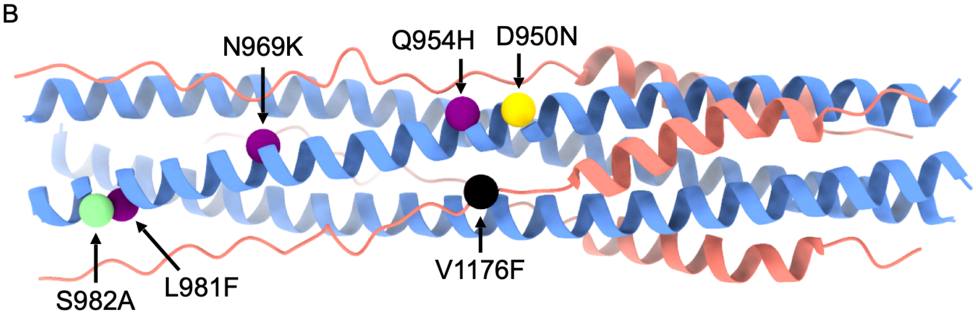

Fig. S8. Mutations of SARS-CoV-2 variants of concern in the HR1HR2 region. (*A*) Sequence alignment of the HR1HR2 region of SARS-CoV-2 variants. (*B*) Locations of mutations in the 3D structure of HR1HR2 (PDB id 8czi). Color code: blue for HR1, red for HR2, green for Alpha, black for Gamma, yellow for Delta, and purple for Omicron.

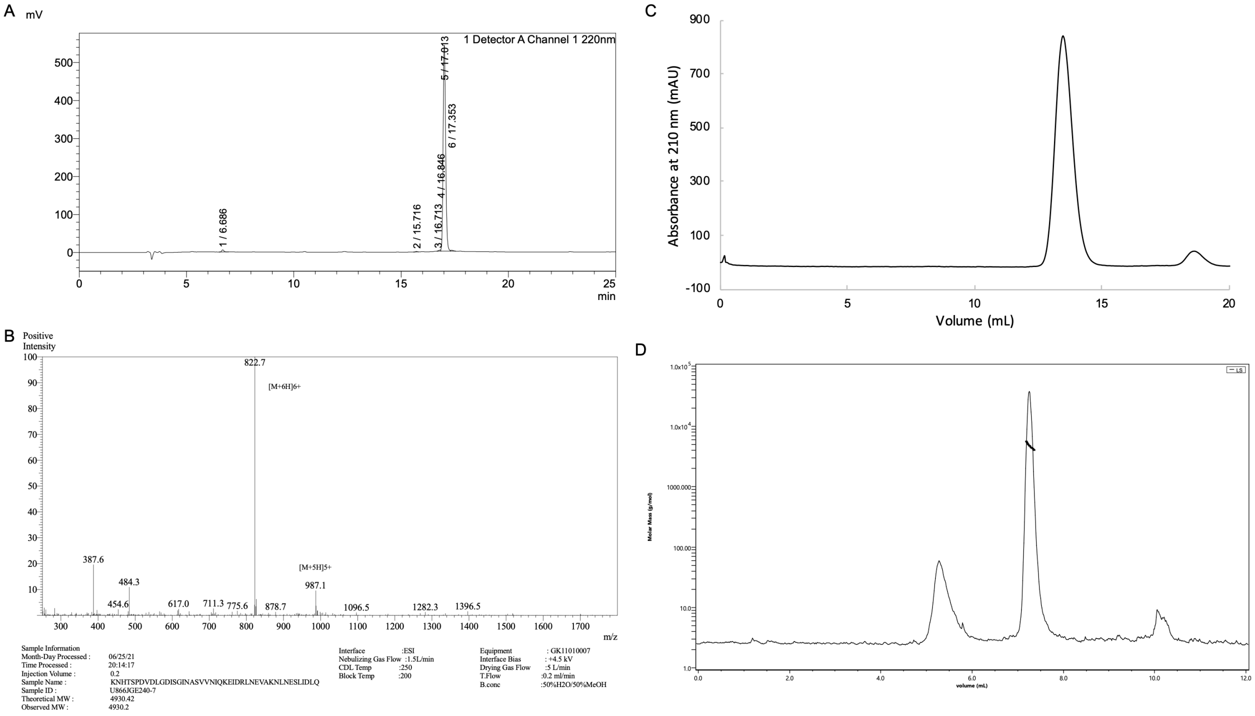

Fig. S9. HPLC (*A*), LC-MS (*B*), SEC (*C*), and SEC-MALS (*D*) profiles of longHR2_45. The molecular weight measured by SEC-MALS is 4.8 kDa, close to 4.9 kDa, the theoretical monomer molecular weight.

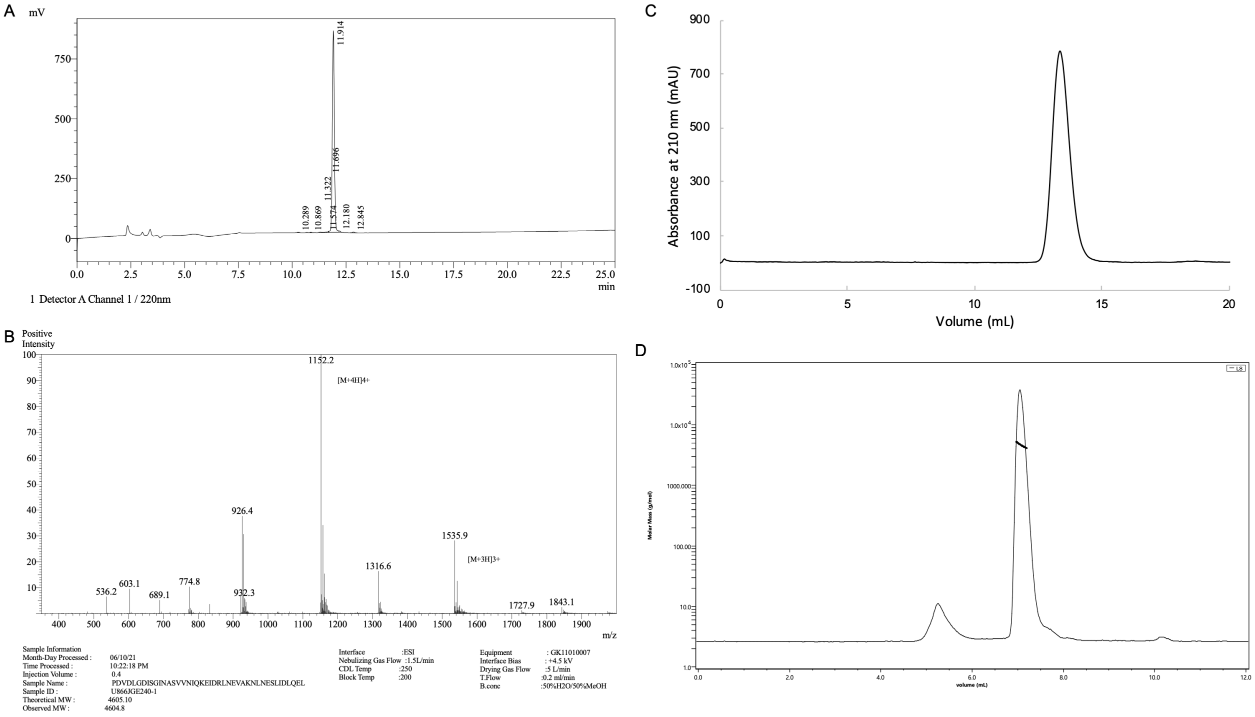

Fig. S10. HPLC (*A*), LC-MS (*B*), SEC (*C*), and SEC-MALS (*D*) profiles of longHR2_42. The molecular weight measured by SEC-MALS is 4.7 kDa, close to 4.6 kDa, the theoretical monomer molecular weight.

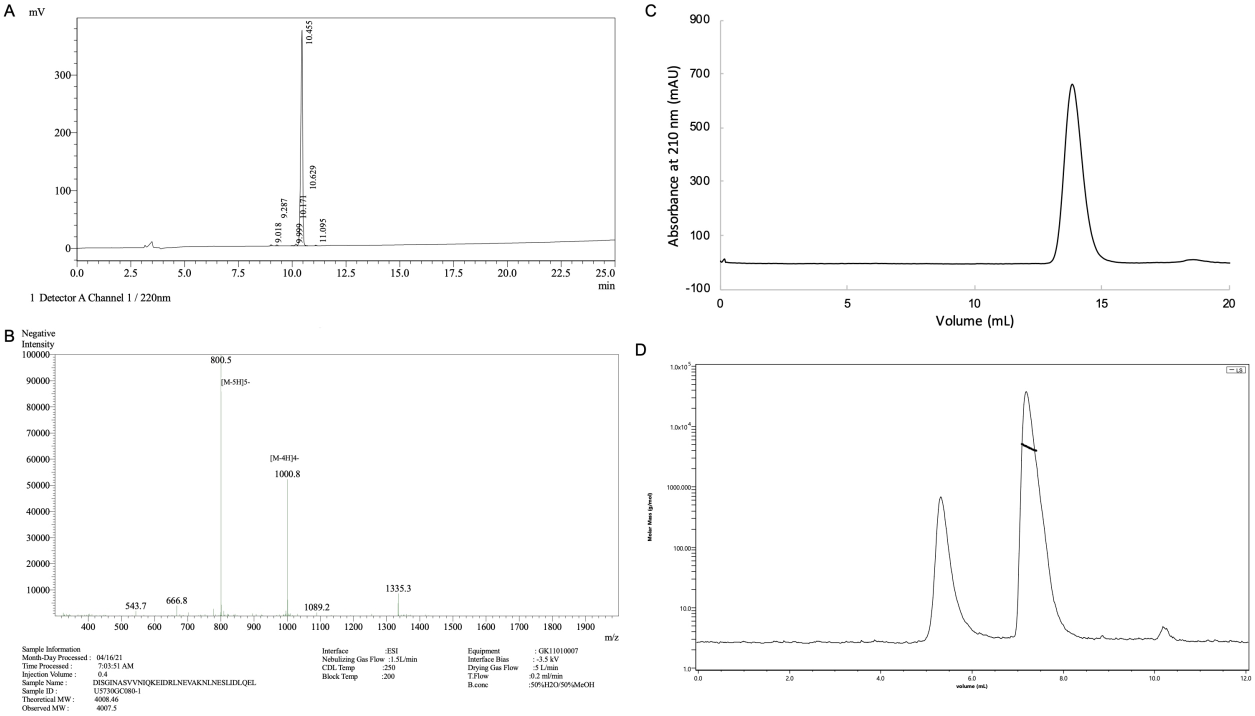

Fig. S11. HPLC (*A*), LC-MS (*B*), SEC (*C*), and SEC-MALS (*D*) profiles of shortHR2. The molecular weight measured by SEC-MALS is 4.5 kDa, close to 4.0 kDa, the theoretical monomer molecular weight.

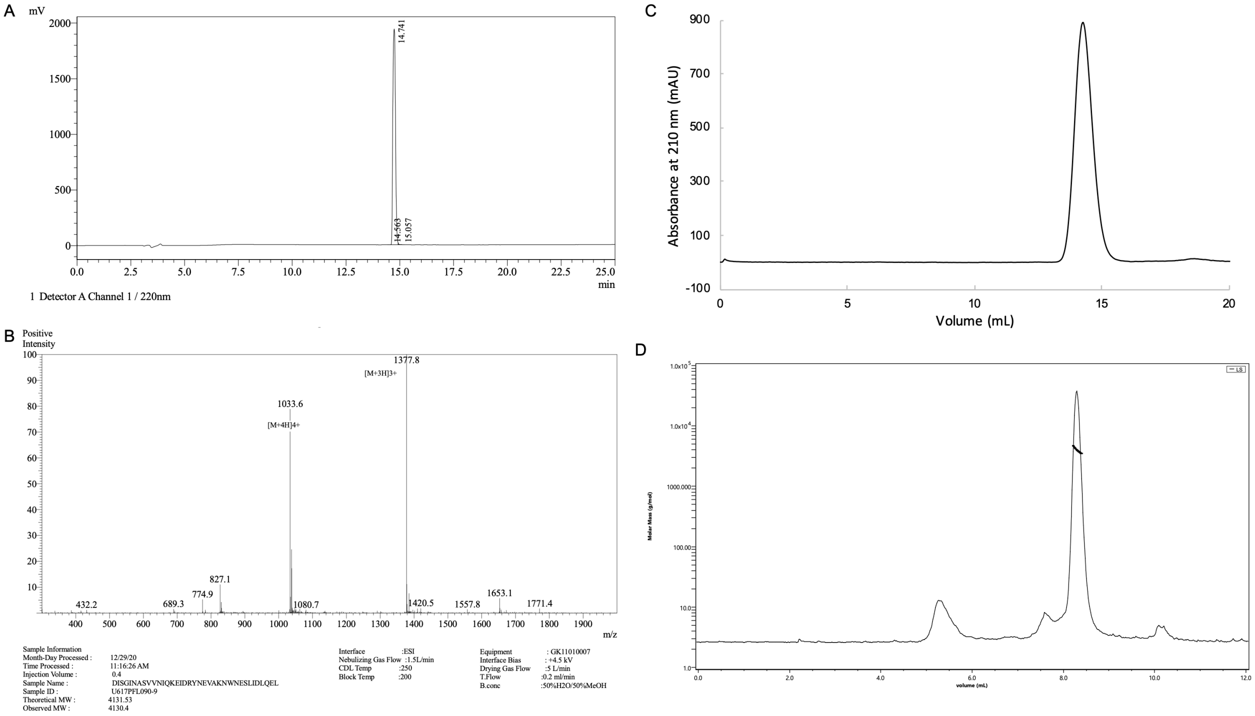

Fig. S12. HPLC (*A*), LC-MS (*B*), SEC (*C*), and SEC-MALS (*D*) profiles of controlHR2. The molecular weight measured by SEC-MALS is 4.0 kDa, close to 4.1 kDa, the theoretical monomer molecular weight.

Table S1. Cryo-EM data collection, refinement, and model building.

	EMDB-27098, PDB 8czi
Microscope	Titan Krios
Voltage (kV)	300
Camera	Gatan K3
Pixel size (Å)	0.653
Exposure time (s)	1
Number of frames per exposure	40
Total Dose ($e^-/Å^2$)	47
Number of movies	18,846
Defocus range (μm)	-2 to -0.3
Number of particles	751,443
Resolution of final global refinement (0.143 FSC, Å)	1.83
Resolution of final local refinement (0.143 FSC, Å)	2.22
Bond RMSD (Å)	0.004
Angle RMSD (°)	0.333
Molprobity score	1.37
Clashscore, all atoms	6.62
Ramachandran favored (%)	100
Ramachandran allowed (%)	0
Ramachandran outliers (%)	0
Rotamer outliers (%)	0
Cβ outliers (%)	0
CaBLAM outliers (%)	0

Table S2. Read counts of variants sequenced during mRNA display.

Variant	0 nM input	10 nM input	32 nM input	100 nM input	0 nM eluate	10 nM eluate	32 nM eluate	100 nM eluate
Scrambled peptide	65952	73405	52473	67097	2501	2765	449	657
shortHR2	2105.19	2726.64	1687.21	2044.18	203.721	618.968	1812.75	2661.66
longHR2_42	17948.6	25428.5	17014.4	22193.4	2156.4	10647.3	30756	44710.5
